# Brain Tumor Discussions on Twitter (#BTSM): Social Network Analysis

**DOI:** 10.2196/22005

**Published:** 2020-10-08

**Authors:** Josemari T Feliciano, Liz Salmi, Charlie Blotner, Adam Hayden, Edjah K Nduom, Bethany M Kwan, Matthew S Katz, Elizabeth B Claus

**Affiliations:** 1 Department of Biostatistics Yale University School of Public Health New Haven, CT United States; 2 OpenNotes Beth Israel Deaconess Medical Center Boston, MA United States; 3 Social Work Hospice Care Team Evergreen Health Medical Center Kirkland, WA United States; 4 Department of Philosophy Indiana University - Purdue University Indianapolis Indianapolis, IN United States; 5 Surgical Neurology Branch National Institute of Neurological Disorders and Stroke National Institutes of Health Bethesda, MD United States; 6 Department of Family Medicine University of Colorado Anschutz Medical Campus Aurora, CO United States; 7 Department of Radiation Medicine Lowell General Hospital Lowell, MA United States; 8 Department of Neurosurgery Brigham and Women's Hospital Boston, MA United States

**Keywords:** brain tumors, social media, health care, patient support, network analysis

## Abstract

**Background:**

The Brain Tumor Social Media (#BTSM) Twitter hashtag was founded in February 2012 as a disease-specific hashtag for patients with brain tumor.

**Objective:**

To understand #BTSM’s role as a patient support system, we describe user descriptors, growth, interaction, and content sharing.

**Methods:**

We analyzed all tweets containing #BTSM from 2012 to 2018 using the Symplur Signals platform to obtain data and to describe Symplur-defined user categories, tweet content, and trends in use over time. We created a network plot with all publicly available retweets involving #BTSM in 2018 to visualize key stakeholders and their connections to other users.

**Results:**

From 2012 to 2018, 59,764 unique users participated in #BTSM, amassing 298,904 tweets. The yearly volume of #BTSM tweets increased by 264.57% from 16,394 in 2012 to 43,373 in 2018 with #BTSM constantly trending in the top 15 list of disease hashtags, as well the top 15 list of tweet chats. Patient advocates generated the most #BTSM tweets (33.13%), while advocacy groups, caregivers, doctors, and researchers generated 7.01%, 4.63%, 3.86%, and 3.37%, respectively. Physician use, although still low, has increased over time. The 2018 network plot of retweets including #BTSM identifies a number of key stakeholders from the patient advocate, patient organization, and medical researcher domains and reveals the extent of their reach to other users.

**Conclusions:**

From its start in 2012, #BTSM has grown exponentially over time. We believe its growth suggests its potential as a global source of brain tumor information on Twitter for patients, advocates, patient organizations as well as health care professionals and researchers.

## Introduction

Social media acts as one of the greatest facilitators of information, ideas, and discussions by creating a borderless global platform. As of April 2019, there are an estimated 3.5 billion active social media users who connect with people around the world at near-instantaneous speed [1]. Three of the largest global platforms are Facebook, WeChat, and Twitter with active user counts of 2.320 billion, 1.098 billion, and 330 million, respectively [2]. Given the ubiquity and integration of social media into everyday life, patients have taken to these platforms to share, connect, collaborate, communicate, and self-create online support communities, making it possible to circumvent traditional barriers to support groups (eg, geography, and lack of time, awareness, and transportation) [3,4].

Twitter makes it possible for unique Twitter users to follow each other, create unique posts, and interact through *tweets*—short messages limited to 280 characters [5]. Tweets can be accompanied by hashtags, which are words preceded by the octothorpe (#) symbol [6]. On Twitter, hashtags become a link that, when clicked, displays other tweets aggregated by the same topic [5,6]. Twitter describes hashtags as a way of categorizing tweets relevant to user’s interest [6]. Recent studies have reported that patients increasingly use disease-specific hashtags as ad hoc support communities to share information and create meaningful connections [7,8]. In more recent years, disease-specific hashtags (including #BTSM [Brain Tumor Social Media hashtag]) have made it possible for clinicians and researchers to connect and communicate with patients and caregivers on areas of shared interest [8,9].

The #BTSM was founded in February 2011 by authors LS and CB [9]—2 brain tumor patients inspired by the success of other Twitter hashtag communities in oncology, notably Breast Cancer Social Media (#BCSM) [10]. LS and CB sought to make it easier for people in the brain tumor community to find each other and connect via Twitter, so they created #BTSM. In a blog post in 2013, LS described #BTSM as a *patient-run* Twitter community *not owned by any organization* [11].

LS and CB started a monthly tweet chat (an organized and moderated, live conversation on Twitter) operating under the Twitter account @BTSMchat in 2013. The #BTSM chat occurs on the first Sunday of every month and discusses broad topics of interest to the brain tumor community (eg, advance care planning, clinical trials, brain tumor representation in arts and entertainment) [11,12]. As of July 2020, @BTSMchat is formally organized by 5 patients, 1 caregiver, and 1 neuro-oncology clinician.

In this study, we examine #BTSM as a hashtag, and its role as an aggregator and technical facilitator of brain tumor conversations by identifying its key users and their interactions, and the type of information shared within this community since 2012. Furthermore, we report recent trends and developments within #BTSM.

## Methods

The data are all publicly available tweets with the #BTSM hashtag from January 1, 2012, to December 31, 2018, obtained via the Symplur Signals platform [13]. Information available includes user names and usage, tweet transcripts, and usage trends over time. Using R (version 3.5.3) [14], we generated descriptive statistics and visual output to provide insights into the overall use (growth) and demographic breakdown (eg, patient, doctor, researcher) of #BTSM users, along with trends and patterns about interactions between users of the #BTSM hashtag. We highlight #BTSM user engagement by describing the proportions of tweets that were retweets, replies, or mentions (ie, tweets that contain another Twitter user’s name). To account for *passive consumption* of tweets featuring the #BTSM hashtag (ie, simply viewing a tweet without engaging with a retweet, reply, or mention), we report total impressions, which is the number of times a tweet has appeared in any user timeline or search result on Twitter [15]. Aggregate data on user engagement were collected to highlight the yearly share of tweet authorship among Symplur-defined user categories. To create a stack plot that focuses on proportions, the yearly raw count for user-specific tweets was transformed to represent yearly share. By representing count as a proportion, the users who generate the most tweets at any given year are presented.

To visualize the interactions between key #BTSM stakeholders, a network plot was created to visualize content sharing via retweets in 2018. Tweet transcripts from Symplur were first downloaded and merged to ensure that all nodes can be plotted and represented in the network. To create the retweet network plot, the open source software Gephi [16] (version 0.9.2) was used. To highlight the users with the most unique connections to other users based on having been retweeted, the color and size of their nodes were adjusted to visualize influential users by applying the outdegree setting on Gephi. Outdegree ranks nodes based on unique outward connections with other nodes [17], which in effect assigned bluer and larger nodes to users with more unique connections. Minor positioning adjustments were made to the network to ensure the readability of nodes and their labels. Additionally, we examined the impact of other hashtags on #BTSM activity in 2018.

## Results

### Content and User Descriptors

**Figure 1:**
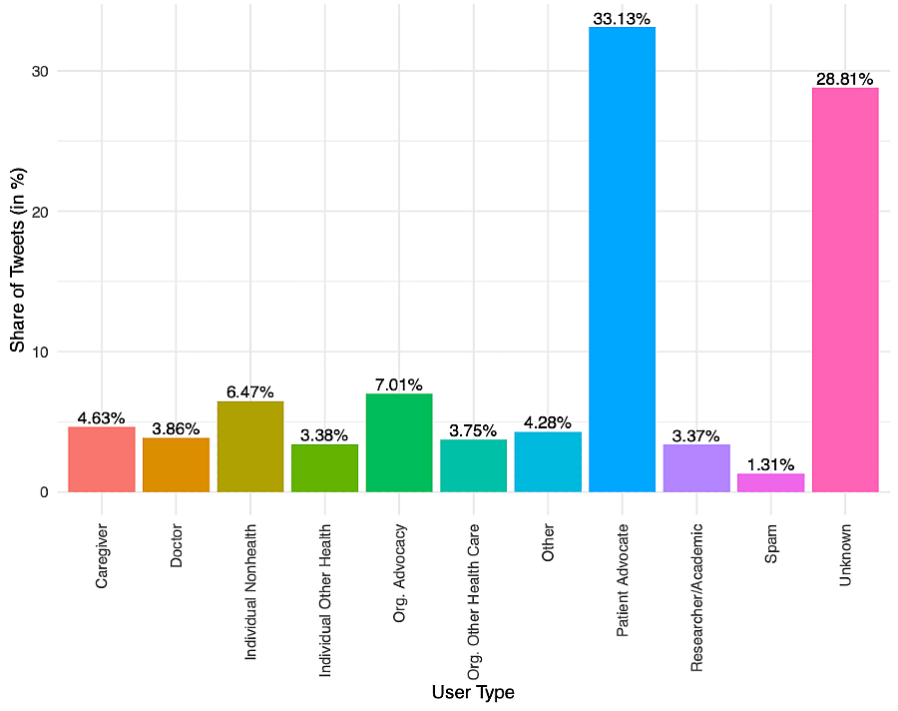
Overall proportion of tweet authorship for 2012-2018.

**Figure 2:**
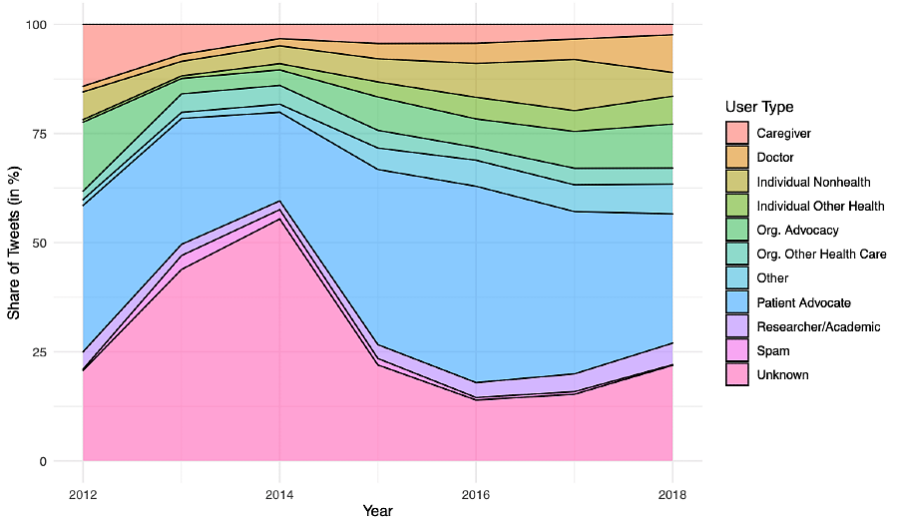
A stacked plot showing the yearly proportion of tweet authorship among users for 2012-2018.

From 2012 to 2018, 59,764 unique Twitter users used the #BTSM hashtag in their tweets, amassing a total of 298,904 tweets (Table 1). Among these, 78.17% (n=233,658), 53.49% (n=159,884), and 5.19% (n=15,516) involved mentions, retweets, and replies, respectively. Many tweets included media, such as website links (42.00%, n=125,534) and media objects such as photos and videos (27.66%, n=82,679). The yearly volume of #BTSM tweets increased by 264.57% from 16,394 in 2012 to 43,373 in 2018, making #BTSM one of the most heavily trended medical hashtags in the Symplur platform. Those identified as patients generated the most #BTSM tweets (33.1%), followed by brain tumor advocacy organizations (28.8%), researchers (7.0%), caregivers (4.6%), and doctors (3.9%; Figure 1). Although still low, the yearly use of #BTSM by doctors has increased over time from 1.3% to 8.6% between 2012 and 2018 (Figure 2). Persons defined as caregivers showed reduced use over time, declining from 14.2% to 2.4%.

**Table 1:** Key #BTSM user and tweet descriptors for 2012-2018.

Data descriptor		Total count
Unique users		59,764
**Tweets generated**		298,904
	Tweets with mentions	233,658
	Tweets that were retweets	159,884
	Tweets with links	125,534
	Tweets with media (photo, video)	82,679
	Tweets that were replies	15,516
Total impression		2,284,771,009

### Hashtags for Expanded Reach

We conducted a deeper analysis on #BTSM user participation and growth for the 2018 calendar year. May 2018 data revealed an increase in #BTSM activity generated by tweets combining both #BTSM and #BTAM, a hashtag created by the National Brain Tumor Society (NBTS) to celebrate the Brain Tumor Awareness Month (BTAM) [18]. Out of the 4620 #BTSM tweets in May 2018, roughly 44% contained #BTAM. Other hashtags combined with #BTSM in May 2018 included #BrainTumor, #BrainTumorThursday, #BrainTumorAwarenessMonth, and #Head2Hill2018 (a public policy advocacy day organized by the NBTS) [19].

In the second half of 2018, our exploratory data analysis found #SNO2018 linked to a mid-November surge in #BTSM activity. The annual meeting of the Society of Neuro-Oncology (SNO) was held in New Orleans, Louisiana, from November 15 to 18, 2018 [20]. The meeting’s official hashtag, #SNO2018, was promoted and used by conference attendees to promote and share information and research at the event. Of the 140 #BTSM tweets in November 15-18, 85 (roughly 61%) also contained #SNO2018, which demonstrates the potential for neuro-oncology professionals to reach a majority of patient and caregiver audience on Twitter.

### Network Influencers

**Figure 3:**
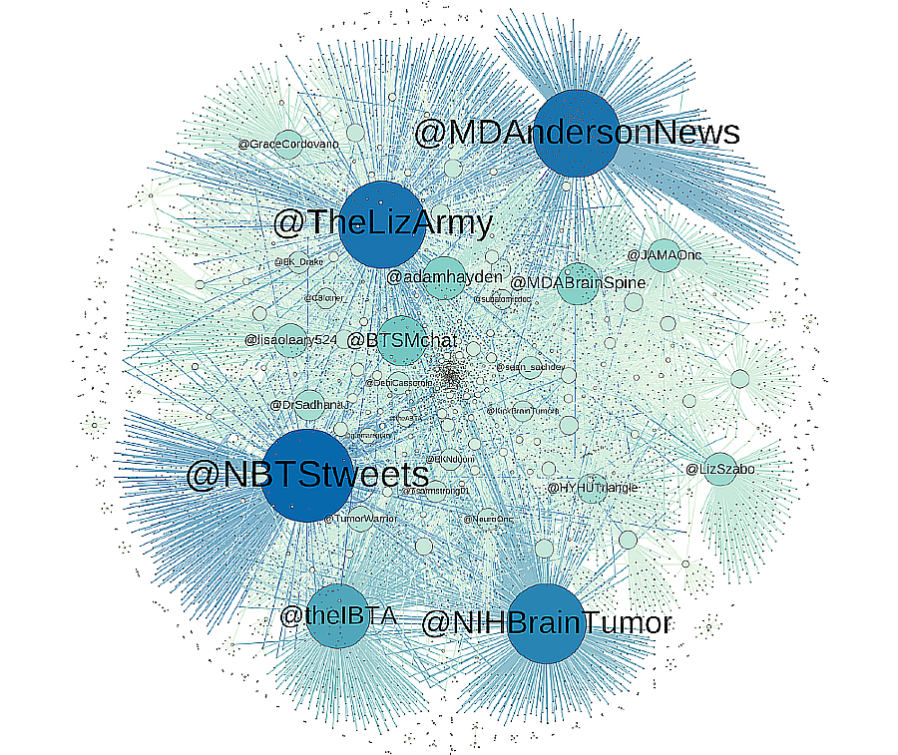
Social networks of most connected #BTSM users in 2018, by retweets.

The 2018 network plot of retweets including #BTSM (Figure 3) identifies a number of key stakeholders from the patient advocate, patient organization, and medical researcher domains and reveals the extent of their reach to other people on Twitter. As indicated by their size and color, 6 of the most connected users in 2018 were @NBTStweets and @theIBTA (patient advocacy organizations), @MDAndersonNews and @NIHBrainTumor (health systems/brain tumor centers), @TheLizArmy (a brain tumor patient), and @BTSMchat (the patient-led community that hosts the monthly #BTSM tweet chats). Further analysis of the 2018 transcripts indicates that a substantial amount of Twitter interactions occurred in the first week of each month, corresponding to the monthly chat hosted by @BTSMchat (Table 2), and includes national and international individuals and organizations.

**Table 2:** Topics Covered by @BTSMchat in 2018.

Month	Topic covered
January	Advance Care Planning
February	N/A^a^
March	Low-Grade Glioma Registry
April	What Does “Quality of Life” Mean to You?
May	Brain Tumor Awareness Month
June	Patient-Driven Symptom Tracking
July	Note to Self: What I Wish I Knew
August	Words Matter: Guilt & the Things We Tell Ourselves
September	Brain Tumors and the Media
October	Patient/Care Partner Relationships
November	Talking About Illness with Family and Friends
December	Breaking Down the Barriers to Clinical Trials

^a^@BTSMchat in February was on break.

## Discussion

This study finds that tweets including the #BTSM hashtag between 2012 and 2018 contained high degrees of Twitter user-to-user engagement where the vast majority of the tweets mentioned at least one other user on Twitter (eg, a reply to a tweet). Although we did not analyze the quality of information shared by users, the study found that sharing content such as website links and media (ie, sharing information) versus text alone corresponds to increased engagement with #BTSM tweets, with 27.66% and 42.00% of the tweets containing visual media (photos/videos) and website links, respectively. Twitter users do not just create unique messages, but also engage with other Twitter users by way of the #BTSM hashtag. As demonstrated in the network plot, major health care organizations (eg, MD Anderson, the National Institutes of Health [NIH], the International Brain Tumour Alliance) now play a central role in #BTSM communication by including the hashtag in their tweets. For future studies, different ways to weigh volume and unique connections, along with user clustering across the network, should be considered to give a more nuanced depiction of the network involved.

A limitation to this study is the focus on metrics that captures participation within #BTSM based on content generation and interaction, which ignores passive Twitter users who might otherwise rely on #BTSM for information without actively engaging through likes and retweets. Twitter users might be searching #BTSM for information without instigating any sort of active and thus trackable method of engagement (eg, tweeting, retweeting, replying, or liking #BTSM tweets). The @BTSMchat community organizers have attempted to track passive viewers during live tweet chats by prompting self-identification through this statement, “If you are here tonight to just listen, please tweet ‘#BTSM’ so we know you’re in the audience” [21]. To the best of our knowledge, we are not aware of any study that has thoroughly examined the extent of passive content consumption within #BTSM or any other disease-specific hashtags. Nevertheless, interaction with the #BTSM hashtag is captured by the total impression count of 2,284,771,009, the tally that any user has been served a content in their timeline or search results.

Throughout the year, major brain tumor events and medical conferences play valuable roles in expanding #BTSM’s reach by introducing the brain tumor community on Twitter to the hashtag. In 2018, attendees and supporters of both BTAM and the Annual Meeting of the SNO heavily utilized #BTSM in their tweets along with other branded hashtags such as #BTAM and #SNO2018. Although it is unclear whether new Twitter users were converted into active #BTSM participants, tweets from any casual user may still be impactful for the awareness they bring to the larger brain tumor community.

One potential limitation for #BTSM growth is the somewhat older age noted for many persons diagnosed with brain tumors. For 2012-2016, the median age at diagnosis for brain and other central nervous cancers was 59 years in the United States [22], although data suggest that the majority of adults in the United States use some form of social media [23]. Among caregivers of patients with childhood and early adolescent cancer, Facebook is the most commonly used social media platform over Twitter and Instagram [24]. Even on Twitter, childhood patient networks are also diffused across multiple hashtags such as #PedOnc (pediatric oncology), #PedPC (pediatric palliative care), #AYACSM (adolescent and young adult cancer social media), and #childhoodcancer. Figure 2 highlights that the proportion of tweets from caregivers has steadily decreased from roughly 14.2% in 2012 to 2.4% in 2018, thereby hinting at much-needed work to integrate patient caretakers into the #BTSM community for support and guidance. The observed decline could be related to the high mortality rate of people with malignant brain tumors, caregiver burnout, and the need to distance oneself from the brain tumor community after the death of a loved one [12]. A proactive method to promote #BTSM participation is to integrate more family members and caretakers to the community, and @BTSMchat organizers recruited a caregiver into their team in 2018. Compared to other disease-specific hashtags, family and caretaker involvement may be particularly important for #BTSM as patients with brain tumor are susceptible to cognitive deficits due to the tumor location.

As #BTSM and other disease-specific hashtags continue to expand, it is increasingly important to address the accuracy of health information shared on Twitter as it affects the public and may have patient safety implications. One way to address these issues is to increase the number of brain tumor stakeholders with a blue Twitter-verified profile badge, which Twitter describes as an authenticity indicator as a public interest account. As of April 1, 2020, the majority of #BTSM influencers identified by our network plot lacks this important badge. By increasing the number of verified accounts, it would be easier for the general public to vet individual accounts they might encounter, thereby increasing the likelihood of accurate information consumption among patients and survivors.

A key feature of #BTSM is the high involvement of national organizations and groups such @BTSMchat in prompting conversations within the hashtag. By hosting a live, facilitated discussion on the first Sunday of each month, @BTSMchat creates an interactive space for various stakeholders within the brain tumor community (eg, patients, caregivers, clinicians, researchers) and forms a structure for sustained engagement. The @BTSMchat chat themes in 2018 ranged from large systemic issues (such as clinical trials and their failure to meet recruitment goals) to more personal topics (such as overcoming guilt, talking with family, holistic patient support through education and peer support, and discussing sensitive topics). @BTSMchat’s identified future goals include (1) widening the lived experiences of tweet chat participants with a focus on social diversity, geographic diversity (rural vs urban), and diversity of central nervous system tumor experience; (2) partnering with moderators of brain tumor Facebook Groups by providing Twitter education and cross promoting the tweet chat; and (3) creating and curating resources for patients and care partners who want to pursue or deepen their participation in peer-to-peer support opportunities, including chat moderation, and through community education, conference attendance, public speaking, and research collaboration through traditional medical research pathways.

When social media platforms first entered the national consciousness, health care professionals could not grasp how these new forms of communication could apply to their work. It is now clear that the larger platforms such as Facebook, WeChat, and Twitter are here to stay. Twitter is a 14-year-old platform that continues to connect people not by personal relationship but rather by shared interests. It is only natural that patients, caregivers, clinicians, and researchers all with a shared interest in neuro-oncology would ultimately converge, share, and form connections, because Twitter was designed to facilitate these forms of information exchange. It is interesting to note that #BTSM was created by patients who saw a needs gap and filled it, without the intervention of a health care structure and is sustained without formal institutional support.

We believe that the growth of the #BTSM hashtag highlights the potential of patient innovation through social media platforms, and serves as a global source of brain tumor information for all brain tumor constituents.

## Abbreviations

BCSM: Breast Cancer Social Media
BTAM: Brain Tumor Awareness Month
BTSM: Brain Tumor Social Media
NBTS: National Brain Tumor Society
NIH: National Institutes of Health
